# Quantitative and qualitative analysis of feedback from The Psychiatry Teaching Programme for Foundation Year doctors rotating through Pennine acute trust from 2010 to 2020

**DOI:** 10.1192/bjo.2021.419

**Published:** 2021-06-18

**Authors:** Angel Namuddu, Margaret Gani, Sarah Burlinson

**Affiliations:** 1 Manchester University NHS Foundation Trust; 2 Search Results Pennine Care NHS Foundation Trust

## Abstract

**Aims:**

To monitor the year on year trend of feedback scores regarding content, presentation and relevance of sessions delivered as part of the programme by analysing the average Likert scales. To review the confidence post topic from FY feedback. To review qualitative data on the written feedback annually using a word cloud.

**Method:**

Collated data from teaching programme from the various teaching sessions from the past decade and analysed previous teaching reports completed by previous ST leads.

**Result:**

Finding: Relevance: Improvement in the average score year on year, highest in 2018/19 at 4.8/5

Content: Improvement in the average score year on year, highest in 2018/19 at 4.6/5.

Delivery: Improvement in the average score year on year, highest in 2018/19 at 4.6/5.

Qualitative analysis showed that the common themes that were commented on as positives for the session were: interactive, relevant and interesting, for areas for improvements the common themes were: more interaction, split into shorter sessions, faster pace and the need practical advice

**Conclusion:**

Recommendations: teaching for FYs should aim to be interactive, relevant and interesting and include practical advice, be shorter and faster paced. Teaching programme organisers to contine to use the foundation year feedback to improve the teaching programme including advising future trainees and organising different topics.

